# Application of a Novel ^68^Ga-HER2 Affibody PET/CT Imaging in Breast Cancer Patients

**DOI:** 10.3389/fonc.2022.894767

**Published:** 2022-05-30

**Authors:** Haitao Miao, Yuyun Sun, Yizi Jin, Xichun Hu, Shaoli Song, Jian Zhang

**Affiliations:** ^1^ Department of Oncology, Shanghai Medical College, Fudan University, Shanghai, China; ^2^ Department of Medical Oncology, Fudan University Shanghai Cancer Center, Shanghai, China; ^3^ Department of Nuclear Medicine, Fudan University Shanghai Cancer Center, Shanghai, China; ^4^ Phase I Clinical Trial Center, Fudan University Shanghai Cancer Center, Shanghai, China

**Keywords:** breast cancer, HER2-positive, PET/CT, ^68^Ga-HER2 affibody, molecular imaging

## Abstract

**Background:**

Breast cancer is a heterogeneous disease, and the human epidermal growth factor receptor 2 (HER2) expression may vary considerably between primary and metastatic lesions, or even within a single lesion. Repeated biopsies cannot always be performed. In this feasibility trial, we assessed whether a novel ^68^Ga-NOTA-MAL-MZHER2 (^68^Ga-HER2) affibody PET/CT could determine the HER2 status of each lesion if there was a clinical need for it.

**Methods:**

^68^Ga-HER2 affibody PET/CT was performed in breast cancer patients if HER2 status remained unclear after standard examinations (including bone scan, ^18^F-FDG PET/CT, CT, and feasible biopsy). All available images for each patient were evaluated through an independent review of two committee-certified radiologists with nuclear medicine expertise. In case of discrepancy, adjudication by a third radiologist was performed as needed. All radiologists were blinded to the clinical information.

**Results:**

Twenty-four patients were enrolled. ^68^Ga-HER2 affibody PET/CT was requested by physicians due to the following reasons: 6 with multiple primary cancers, 13 with metastases not amenable to biopsy or repeated biopsy, 6 with inconsistent HER2 status between primary and metastatic lesions, and 4 with different HER2 status within different metastases. The final PET report revealed that the ^68^Ga-HER2 affibody tumor uptake was considered positive in 16 patients, negative in 7 patients, and equivocal in one patient. The heterogeneity of ^68^Ga-HER2 affibody uptake was observed, with a maximal 8.5-fold difference within one patient and a maximal 11-fold difference between patients. ^68^Ga-HER2 affibody PET/CT demonstrated a high diagnostic accuracy in differentiating HER2-enriched breast cancer, with a sensitivity of 91.7% and a specificity of 84.6%, regardless of prior lines of anti-HER2 therapies.

**Conclusion:**

^68^Ga-HER2 affibody PET/CT imaging could provide valuable information on HER2 expression of each tumor in the body of patients, which may help in personalized clinical decision-making. Its value is now under systemic assessment.

## Introduction

Approximately 15%–30% of invasive breast cancer cases demonstrate human epidermal growth factor receptor 2 (HER2) overexpression and/or gene amplification, which is associated with aggressive biological behavior and an unfavorable clinical outcome (1). At the same time, HER2 also serves as a critical therapeutic target in breast cancer, and its status has so far been acknowledged as the best biomarker for predicting response to anti-HER2 therapies. Therefore, the accurate assessment of HER2 expression in breast cancer is essential for clinical diagnosis and treatment decisions.

Current diagnosis of HER2 overexpression or gene amplification relies on the immunohistochemistry (IHC) and/or *in situ* hybridization (ISH) tests on pathological specimens. However, the spatiotemporal heterogeneity of HER2 expression poses a challenge to the determination of HER2 status in breast cancer using biopsies. The HER2 status in metastases has been observed to be different from that of the primary breast cancer in 3%–10% of patients, or to be even inconsistent within a single lesion (2–5). In addition, HER2 status may sometimes change during anti-HER2 treatment, which can be partially explained by the drug sensitivity of HER2-positive tumor cells (6, 7). These results indicate the necessity of HER2 re-evaluation for breast cancer patients during the disease course, especially for recurrent diseases. However, repeated biopsies are not always clinically feasible in patients. Therefore, alternative noninvasive modalities with high accuracy are urgently needed to determine HER2 status in breast cancer.

Molecular imaging serves as a promising alternative to monitor whole-body HER2 expression quantitatively and dynamically during the management of breast cancer. Radiolabeled HER2 antibody, antibody fragments, and affibody are representative HER2-targeted imaging modalities under current clinical investigations. Positron emission tomography (PET) using HER2 antibodies labeled with radionuclides such as ^111^In, ^64^Cu, and ^89^Zr demonstrates sensitive and specific HER2 uptake in HER2-positive metastatic and primary breast cancer, and it can also identify unsuspected HER2-positive lesions (8–11). However, their clinical use was limited by disadvantages such as slow pharmacokinetics and relatively high radiation dosage. HER2 antibody fragment labeled with ^68^Ga is well-tolerated and allows imaging in a shorter time after injection, but shows a lower sensitivity of lesion detection (12). Affibody molecules are a group of small and robust affinity proteins (6.5 kDa) engineered to mimic antibody based on the immunoglobulin G domain of staphylococcal protein A (13, 14). Due to its higher sensitivity and rapid penetration into target lesions (15, 16), affibody demonstrates superiority in molecular imaging compared with monoclonal antibody and antibody fragments, especially in the detection of metastases inaccessible to biopsies. Affibody also requires lower effective doses and can be rapidly eliminated from blood, which yields a better tolerance and safety profile than other affinity proteins. ^68^Ga-HER2 affibody PET/computed tomography (CT) is one of its imaging applications that has been supported by preclinical and clinical evidence (17–19). ^68^Ga-HER2 affibody PET/CT indicates a high accuracy in the detection of converted HER2 expression in metastatic cancer, which may assist in anti-HER2 treatment modification during patient management (20, 21).

In this prospective pilot study, we aimed to evaluate the clinical value of ^68^Ga-HER2 affibody PET/CT to noninvasively assess the HER2 intensity of lesions using pathologically confirmed HER2 status as the standard and to then explore its value in clinical decision-making regarding the use or non-use of anti-HER2-targeted agents in patients who were otherwise inappropriate candidates for routine use of anti-HER2 agents.

## Methods and Materials

### Study Participants

All patients were histopathologically confirmed with breast cancer. ^68^Ga-HER2 affibody PET/CT scan was requested in breast cancer patients with HER2 status that needed to be determined in any tumor in the body of patients after standard examinations (including bone scan, ^18^F-fluorodeoxyglucose [^18^F-FDG] PET/CT, CT, and feasible biopsy), although there had been results of HER2 status or patients with metastases not feasible for biopsy or repeated biopsy. Informed consent had been signed by all patients before study enrollment. All clinical information was retrieved from case records. This study was done in Fudan University Shanghai Cancer Center from June 2020 to January 2021. Patients used anti-HER2-targeted agents based on results of ^68^Ga-HER2 affibody PET/CT.

### Radiosynthesis and PET/CT Imaging Protocol

^68^Ga-HER2 affibody was produced in conformity with previous literature (17). Scanning was performed on a Siemens Biograph 16HR PET/CT scanner. Before administering 3.0 MBq/kg (0.08 mCi/kg) ^68^Ga-HER2 affibody intravenously, all patients were asked to fast for at least 4 h to reduce gastrointestinal uptake. Patients were requested to keep quiet before and after injection of the tracer. About 2 h after the administration, whole-body PET/CT scanning was initiated. The scanning began with low-dose CT (120 kV, 250–300 mA, pitch 3.6, and rotation time 0.5). A PET examination scan (16.2 cm axial field width, 2–3 min per table position) was obtained immediately after CT scanning. The attenuation-corrected PET data were reconstructed iteratively by the standardized ordered-subset expectation maximization (OSEM) technique and were reoriented in axial, sagittal, and coronal sections.

### Image Interpretation and Uptake Calculation

Two experienced nuclear medicine physicians independently analyzed and interpreted the images blindly, and they reached a consensus in case of inconsistency. For quantitative analysis, a multimodality computer platform (Syngo, Siemens, Knoxville, Tennessee, USA) was used. The maximum standardized uptake value (SUV_max_) normalized to body weight was calculated by drawing a 3-dimensional volume of interest with each lesion.

### Immunohistochemistry/Fluorescence *In Situ* Hybridization Assays for Deciphering HER2 Status

HER2 immunohistochemistry (IHC) tests were conducted and interpreted according to the most updated American Society of Clinical Oncology (ASCO)/College of American Pathologists (CAP) Clinical Practice Guideline (22). Tumors with IHC 0 or 1+ were interpreted as HER2-negative, and tumors with IHC 3+ were interpreted as HER2-positive. Specimens with IHC 2+ would be further assessed with fluorescence *in situ* hybridization (FISH) in the pathology department of our hospital and finally determined based on combined interpretation of the IHC and FISH assays.

### Statistical Analysis

The quantitative data in compliance with normal distribution were presented with mean SUV_max_± standard deviation. The objective response rate (ORR) was defined as the percentage of patients with complete response or partial response after being given the anti-cancer treatment. Progression-free survival was calculated as the time from the initiation of the anti-cancer treatment to disease progression or death from any cause. All the lesions were assessed based on the Response Evaluation Criteria in Solid Tumors (RECIST) 1.1.

## Results

### Patient Characteristics

Twenty-four patients were enrolled from June 2020 to January 2021 in Fudan University Shanghai Cancer Center. Patient characteristics are summarized in **Table 1**. In total, 24 patients were enrolled in this study. Of all the patients, 15 had received anti-HER2 treatment before. ^68^Ga-HER2 affibody PET/CT scan was requested by physicians due to the following reasons (**Table S1**): (a) to differentiate among metastases of multiple primary cancers, including either patients who harbored two primary breast malignancies but with different HER2 status or patients who had at least another non-breast primary other than HER2-positive breast cancer (*n* = 6); (b) to assess the HER2 status of a single lesion not amenable to biopsy or, in case of multiple lesions, inaccessible to repeated biopsy (*n* = 13); (c) to evaluate inconsistent HER2 status between the primary and metastatic lesions (*n* = 6); and (d) to assess different HER2 status within metastases (IHC ranging from 0 to 3+) (*n* = 4).

**Table 1:** Patient characteristics.

Characteristic	All female patients (*N* = 24)
Median age, years (range)	50 (30–71)
Prior lines of anti-HER2 therapy	
0	9
1	9
2	4
≥3	2
Reasons for selection of the test	
Synchronous multiple primary cancers	6
Inaccessibility for (repeated) biopsy	13
HER2 discordance between primary and metastatic lesions	6
HER2 discordance between different metastatic lesions	4

### Sensitivity and Specificity of ^68^Ga-HER2 Affibody PET/CT

All 24 patients underwent ^18^F-FDG PET/CT and ^68^Ga-HER2 affibody PET/CT. Patients were monitored at 1 h, 2 h, and the following day after the tracer injection, and no side effects were observed or reported. Heterogeneity of tumor tracer uptake was observed within patients, with a maximal 8.5-fold difference within one patient (SUV_max_ from 3.4 to 28.8). Also, tumor tracer uptake varied greatly between patients, with a maximal 11-fold difference (SUV_max_ from 2.6 to 28.8).

All patients had at least one site of metastatic disease proven by biopsy. For central revision, a total of 37 tumor samples of 24 patients were available. Pathological results of HER2 status of each punctured lesion by IHC/FISH were used as the standard. In 24 HER2-positive tumors, 22 tumors were also positive with a sensitivity of 91.7%. In 13 HER2-negative tumors, 11 tumors were also negative with a specificity of 84.6%.

### ^18^F-FDG PET/CT and ^68^Ga-HER2 Affibody PET/CT

The highest ^68^Ga-HER2 affibody uptake of normal organs was observed in the kidney (SUV_max_ = 46.2), followed by the liver, small intestine, thyroid gland, spleen, left ventricular wall, colon, uterus, stomach, blood pool, lung, and the bone cortex, with the lowest being seen in the bone marrow and the brain (SUV_max_ = 0.76) (**Figure S1**).

An assessment report showed that ^68^Ga-HER2 affibody tumor uptake was considered positive in 16 patients, negative in 7 patients, and equivocal in one patient (**Table S1**). The most common sites of metastases were lymph nodes (*n* = 14) and bone (*n* = 14), followed by liver (*n* = 10), lung (*n* = 6), chest wall (*n* = 5), brain (*n* = 2), thyroid (*n* = 1), and under the skin (*n* = 1). Seventeen of 24 patients had multiple organ involvement.

A total of 293 tumor lesions were identified in ^68^Ga-HER2 affibody PET after primary visual assessment, of which 177 (60.4%) were considered measurable. In 4 patients, none of the known metastases appeared on ^68^Ga-HER2 affibody PET, which was confirmed in ^18^F-FDG PET/CT scans. In the remaining 20 patients, a median of 9 lesions (range 1–62) was measurable.

### Clinical Value of ^68^Ga-HER2 Affibody PET

**Figure 1:**
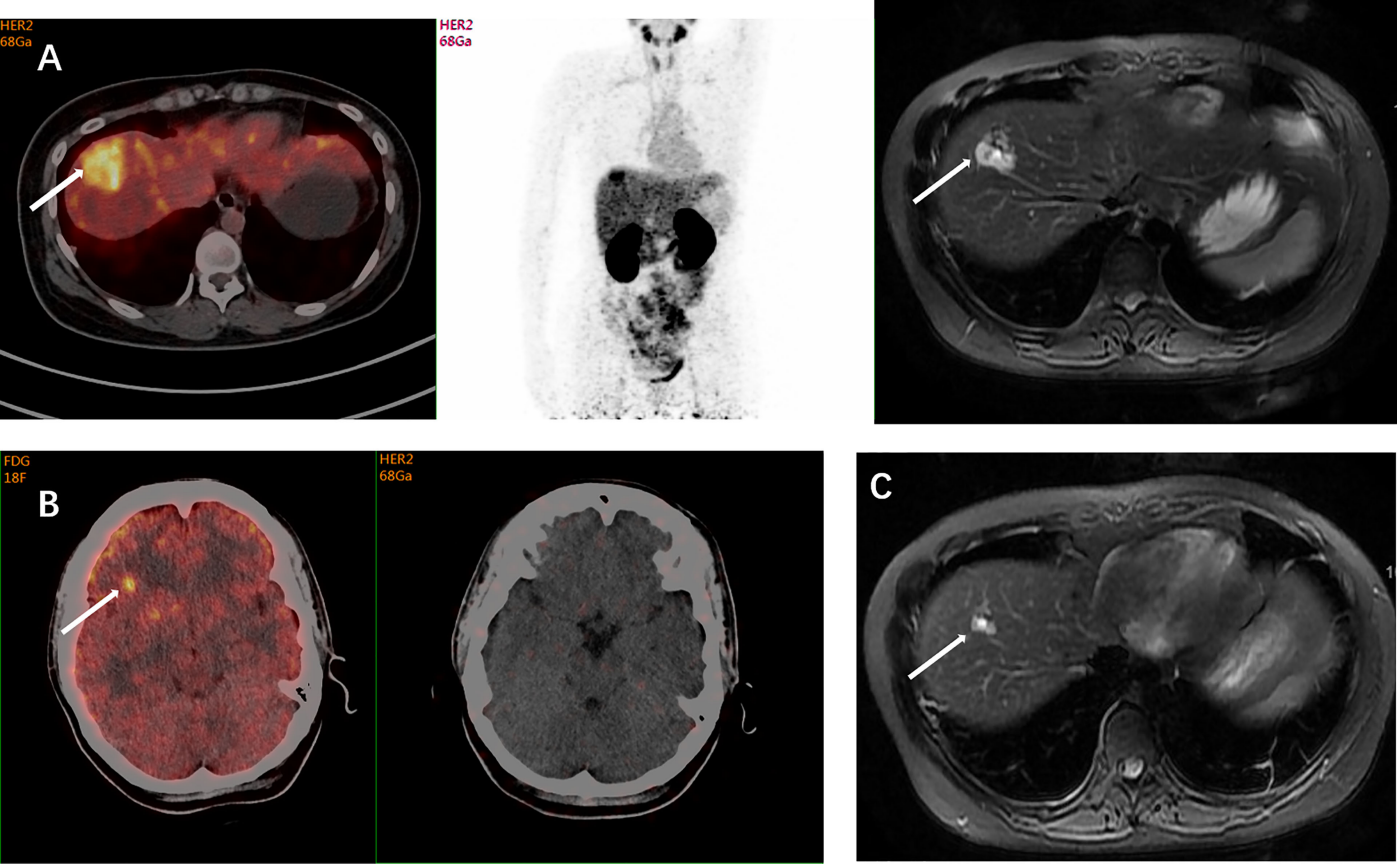
A 39-year-old woman was diagnosed with ER-positive/HER2-negative breast cancer. The histopathological type of the primary tumor was mucinous adenocarcinoma accompanied by intraductal carcinoma. Three years after the mastectomy, suspicious metastases were detected in her left lung and right liver during the follow-up examinations. The IHC test indicated a HER2-negative (1+) lesion in her left lower lobe in another hospital, but showed IHC 2+ with a positive HER2 FISH result in our hospital. The inconsistent results of HER2 status implicated the spatiotemporal heterogeneity of HER2 expression, and the HER2 status of liver metastases remained unclear. FDG-PET showed FDG avidity in the right lobe of liver and focal FDG avidity in the right frontal lobe. Axial MRI and ^68^Ga-HER2 affibody PET/CT demonstrated ^68^Ga-HER2 affibody avidity in the right lobe of the liver (arrow, SUV_max_ of 10.4) **(A)** and no ^68^Ga-HER2 affibody avidity in suspicious frontal lobe lesions **(B)**. The HER2 positivity of the lesion in the right lobe of the liver on affibody PET was later confirmed pathologically by puncture biopsies. Based on the results of HER2 PET and pathology, systemic anti-HER2 treatment was initiated in this patient including trastuzumab and pertuzumab. Her follow-up axial MRI after 2 months of treatment demonstrated resolution of nodes, which indicated that the patient is responsive to anti-HER2 treatment **(C)**. This case showed the value of ^68^Ga-HER2 affibody PET in the HER2 status determination and treatment decision-making.

**Figure 2:**
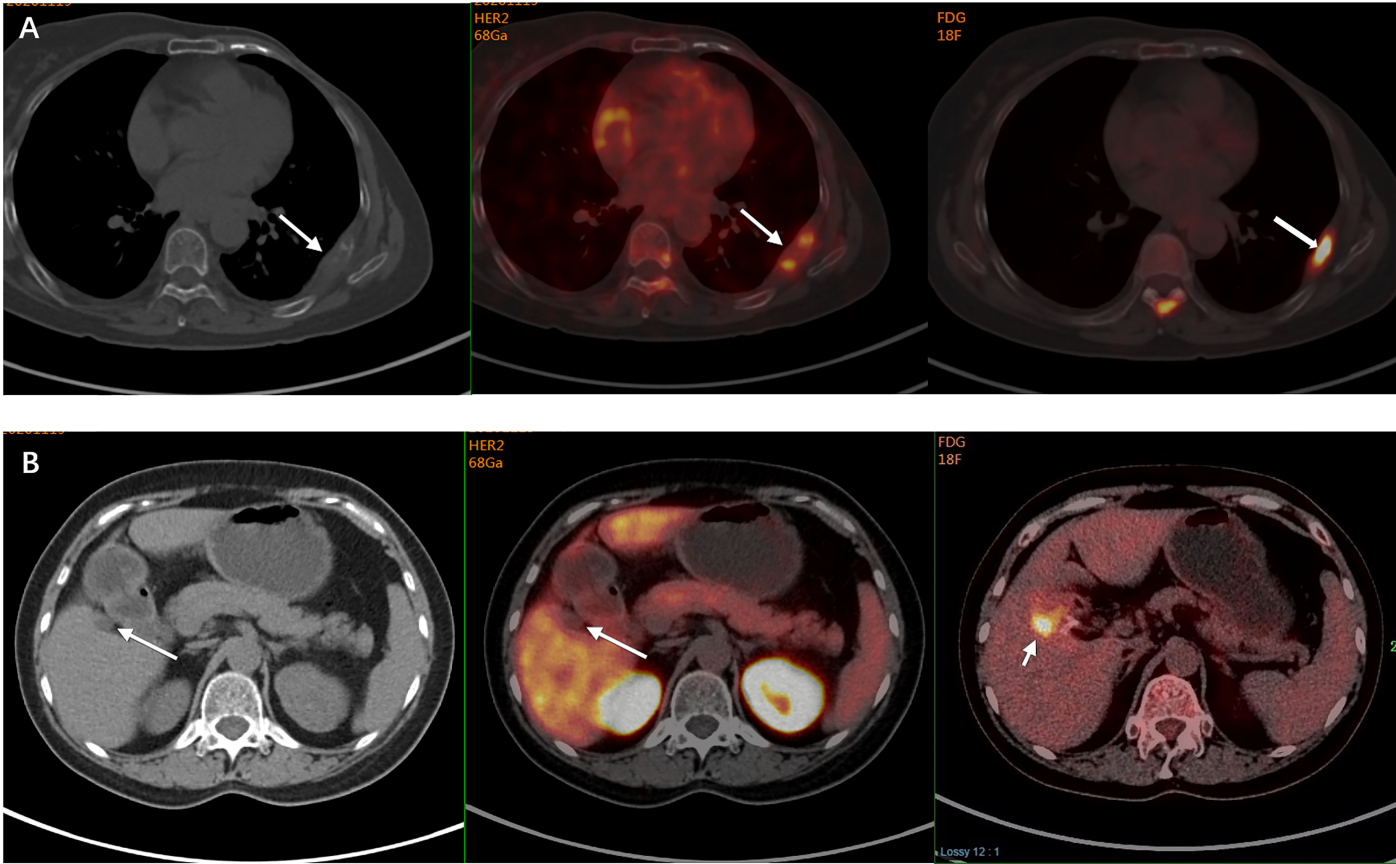
A 60-year-old woman was diagnosed with primary ER-negative/HER2-positive invasive ductal breast carcinoma. Two years later, she developed bone metastases in her left posterior ribs. PET/CT also demonstrated a hypodense shadow in the right anterior lobe of the liver with increased FDG uptake on the FDG PET, and a liver metastasis was suspected. Subsequent ^68^Ga-HER2 affibody PET/CT showed that the HER2 uptake in the left posterior rib was increased (arrow, SUV_max_ of 4.4) **(A)**, while no ^68^Ga-HER2 affibody avidity was found in the right anterior lobe of her liver close to the gallbladder fossa though increased FDG uptake was shown in the same site (arrow) **(B)**. The pathology of liver biopsy revealed that the suspicious lesion in her liver was actually the infiltration of inflammatory cells in the liver portal area. The manifestation on ^68^Ga-HER2 affibody PET/CT scan was consistent with the pathology, which suggested the extended use of HER2 PET in the discrimination of metastases.

Five patients were reclassified as HER2-positive after the detection and all received anti-HER2 treatment with an ORR of 60% (3/5) and a median PFS of 11.8 months (**Table 2**). The patient in **Figure 1** was one of the five cases. Two patients were reclassified as HER2-negative after the detection and both received treatment without anti-HER2 agents, one of whom had an objective response. **Figure 2B** shows the increased uptake of FDG in the right anterior lobe of the liver and no ^68^Ga-HER2 affibody avidity. **Figure 2A** demonstrates that the HER2 uptake increases in the metastatic tumor in left posterior rib. The pathology demonstrated inflammation in liver biopsy.

**Table 2:** Treatment decision changes before and after ^68^Ga-HER2 affibody PET/CT.

Planned treatment	Treatment given after ^68^Ga-HER2 affibody PET/CT	
	Anti-HER2 (± chemotherapy)	No anti-HER2 treatment
Anti-HER2 (± chemotherapy)	15	2
No anti-HER2 treatment	5	2

## Discussion

In this prospective study, ^68^Ga-HER2 affibody PET/CT demonstrated a high diagnostic accuracy in differentiating HER2-enriched breast cancer, with a sensitivity of 91.7% and a specificity of 84.6%, regardless of prior lines of anti-HER2 therapies. It is a clinically feasible modality for the whole-body evaluation of HER2 status noninvasively and may complement the current standard HER2 testing approach. Small malignant lesions could not be missed, because they can be found by the morphologic CT component of the PET/CT. In the present study, ^68^Ga-HER2 PET/CT showed a similar performance with ^18^F-FDG in assessing metastases. Xu and his colleagues investigated both ^18^F- and ^68^Ga-labeled HER2 affibody and found that both of the radiotracers displayed specific binding to the HER2 receptor. However, ^68^Ga-HER2 affibody could be easily prepared in nearly 20 min with good radiochemical purity. Compared with the ^18^F-labeled counterpart, the yields and specific activity of ^68^Ga-HER2 affibody were both significantly increased (~80% vs. ~10% and ~23 GBq/μmol vs. ~9 GBq/μmol, respectively) (17). Multiple synthesis steps and low labeling yields may partially limit the use of ^18^F-HER2 affibody.

For breast cancer patients who develop recurrent or metastatic lesions, accurate HER2 evaluation is vital for the determination of optimal treatment strategies. Previous studies have found out the spatiotemporal heterogeneity of HER2 expression between or within primary and metastatic tumors, which was also verified in this study. On this account, HER2 re-evaluation of recurrent and metastatic sites has been highly recommended by the ASCO/CAP guideline, even for patients with HER2-negative primary breast cancer (23). Histopathological evaluation based on tissue biopsy is the current standard testing for HER2 status, while repeated biopsy is not always possible, especially for metastatic lesions in locations difficult for biopsies, such as the brain, lung, sternum, and adrenal gland. HER2 PET/CT demonstrates its advantage as a noninvasive modality to detect HER2-positive lesions based on the whole-body scan in these settings.

Higher radioactivity accumulation was found in HER2-high-expressing BT474 cells/xenografts than in HER2-lower-expressing MCF-7 cells/xenografts. Additionally, one breast cancer patient with a HER2 IHC score of 3+ had a higher SUV_max_ than the patient with a score of 1+ (17). Other previous studies described a good relationship between the FISH ratio and SUV_max_, and a significant linear correlation between the HER2 affibody uptake value and relative HER2 expression levels was also found (21, 24). These results implied that the probe might have the capability to accurately diagnose HER2 levels. The surveillance of HER2 status using ^68^Ga-HER2 affibody PET can also provide useful information for patients’ prognosis and treatment modification. Both retrospective and prospective studies have shown that HER2 status can possibly change during anti-HER2 treatment (7, 25, 26). A higher proportion of HER2 discordance is found in patients after neoadjuvant chemotherapy alone than after neoadjuvant chemotherapy combined with anti-HER2 treatment. Loss of HER2 positivity has been reported to be associated with significantly worse disease-free survival and overall survival compared with those with maintained HER2 positivity in breast cancer patients (27, 28). In addition, data have shown that patients who gain HER2 positivity during progression can derive extra benefit from anti-HER2 treatment in the metastatic setting (29). Meanwhile, ^68^Ga-HER2 affibody PET imaging will not be affected by anti-HER2 treatment, since HER2 affibodies bind with different extracellular domains of HER2 from HER2 antibodies. Therefore, ^68^Ga-HER2 affibody PET/CT may be useful as an individualized “image and treat” strategy for the monitoring of changes in receptor expression during treatment, which may facilitate clinicians to optimize treatment decisions promptly.

Our study also suggested the expanded clinical value of ^68^Ga-HER2 affibody PET/CT in the differential diagnosis of breast cancer metastases and synchronous primaries based on the determination of HER2 status. ^18^F-FDG PET/CT has been extensively used to detect suspicious multiple metastases of the whole body, and can sometimes identify unexpected lesions, but it cannot always distinguish well between inflammatory lesions and metastases since both may demonstrate high metabolic activity characterized by increased glucose uptake on the scan. The HER2 uptake of suspicious lesions on ^68^Ga-HER2 affibody PET/CT scan can provide an important clue for the diagnosis of metastases from HER2-positive breast cancer and discriminate from inflammation. Previous studies have reported that approximately 12% of breast cancer survivors are diagnosed with second malignant primaries (30), and the incidence of occurring multiple primaries ranges from 4.1% to 16.4% (31). It is critical to discriminate synchronous second or multiple primaries from metastatic lesions in patients with breast cancer since their therapeutic principles are totally different, while invasive diagnostic procedures are always needed in these cases. In this study, a different HER2 uptake was shown on the HER2 PET/CT scan in synchronous primaries confirmed by clinical or pathological diagnosis. ^68^Ga-HER2 affibody PET/CT can serve as a noninvasive approach to assess the HER2 expression of multiple lesions simultaneously, which may extend its use in facilitating the clinical diagnosis of synchronous primaries.

There are also several noteworthy limitations. First, the sample size for analyzing diagnostic accuracy was relatively small. Further validations of its diagnostic ability in a larger population are needed. On top of that, the cutoff values of ^68^Ga-HER2 affibody PET/CT for discriminating HER2-positive breast cancer and defining HER2 low expression are yet to be defined. We look forward to deriving the cutoff values of ^68^Ga-HER2 affibody PET/CT for discriminating HER2-positive/negative breast cancer from further clinical trials. Two clinical trials of ^68^Ga-HER2 affibody PET/CT are currently ongoing in our center: one trial evaluates its ability to differentiate a HER2-enriched subtype of metastatic breast cancer with an exploration of cutoff value (STANDPOINT, NCT: 04758416), and the other trial investigates its predictive value in anti-HER2 treatment through dynamic surveillance (DOLPHIN, NCT: 04769050).

## Data Availability Statement

The original contributions presented in the study are included in the article/**Supplementary Material**. Further inquiries can be directed to the corresponding authors.

## Ethics Statement

The studies involving human participants were reviewed and approved by the Ethics Committee of Fudan University Shanghai Cancer Center. The patients/participants provided their written informed consent to participate in this study. Written informed consent was obtained from the individual(s) for the publication of any potentially identifiable images or data included in this article.

## Author Contributions

HM, XH, SS, and JZ contributed to the conceptualization and design of the study. HM and YS were responsible for the collection and assembly of data. YJ, YS, and HM completed the statistical analyses and drafted the manuscript together. XH, SS, and JZ revised the manuscript with constructive ideas. All authors contributed to the article and approved the submitted version.

## Funding

This study was supported by the National Natural Science Foundation of China (grant no. 82072915); Project of Shanghai Municipal Health Commission (grant no. 202140397); CSCO-ROCHE Cancer Research Fund 2019 (grant no. Y-2019Roche-171); and Chinese Young Breast Experts Research project (grant no. CYBER-2021-001).

## Conflict of Interest

The authors declare that the research was conducted in the absence of any commercial or financial relationships that could be construed as a potential conflict of interest.

## Publisher’s Note

All claims expressed in this article are solely those of the authors and do not necessarily represent those of their affiliated organizations, or those of the publisher, the editors and the reviewers. Any product that may be evaluated in this article, or claim that may be made by its manufacturer, is not guaranteed or endorsed by the publisher.
