# Supplementary material for: Application of a Novel 68Ga-HER2 Affibody PET/CT Imaging in Breast Cancer Patients
Source: Front Oncol. 2022 May 30;12:894767. doi: 10.3389/fonc.2022.894767 (PMC9195516; doi:10.3389/fonc.2022.894767)
Supplement: Supplementary file 1 [file DataSheet_1.docx]

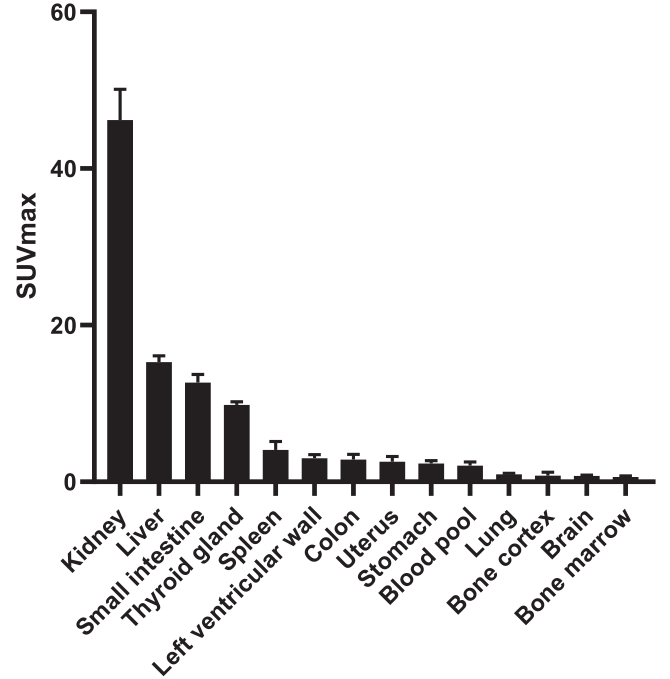


**Fig S1** ^68^Ga-HER2 uptake distribution in the normal organs depicted as mean SUVmax (+SD)

Table S1: Details on the clinical dilemma and the results per scan modality per patient

| Patient | Dilemma category | Description of dilemma | Sites of Malignancy  at Enrollment by Standard Examinations | ^18^F-FDG PET/CT positive lesions | ^68^Ga-HER2 affibody PET/CT suspicious positive lesions | ^68^Ga-HER2 affibody PET/CT interpretation |
| --- | --- | --- | --- | --- | --- | --- |
| 1 | Heterogeneous HER2 status over time | HER2- primary breast cancer and HER2+ metastasis during course of disease | Liver, lung | Liver | Liver | Lung metastasis have been removed, liver metastasis shows ^68^Ga-HER2 affibody uptake, scan considered positive |
| 2 | Heterogeneous HER2 status over time | HER2- primary breast cancer and HER2+ metastasis during course of disease | Liver, lung | Liver | Liver | Lung metastasis have been removed, liver metastasis shows ^68^Ga-HER2 affibody uptake, scan considered positive |
| 3 | Heterogeneous HER2 status during time | Different HER2-status within metastases during course of disease | Chest wall, nodes | Chest wall, breast, nodes | Chest wall, breast, nodes | Dominant part of tumour load shows ^68^Ga-HER2 affibody uptake, scan considered positive |
| 4 | Evaluation of HER2 status | Suspicious lymph node, no biopsy possible | Nodes | Nodes | Nodes | Lymph node metastasis shows ^68^Ga-HER2 affibody uptake, scan considered positive |
| 5 | Heterogeneous HER2 status over time | HER2- primary breast cancer and HER2+ metastasis during course of disease | Liver, bone, nodes | Liver, bone, nodes | Liver, bone, nodes | Dominant part of tumour load shows ^68^Ga-HER2 affibody uptake, scan considered positive |
| 6 | Evaluation of HER2 status | Multiple lesions, no repeated biopsy possible | Liver, nodes | Nodes | Nodes | Lymph node metastasis shows ^68^Ga-HER2 affibody uptake, no 18F-FDG and ^68^Ga-HER2 affibody uptake in liver lesion, scan considered positive |
| 7 | Evaluation of HER2 status | Suspicious lymph node, no biopsy possible | Sternum, nodes | Sternum, nodes | Sternum | Sternum metastasis shows ^68^Ga-HER2 affibody uptake, remaining lesions don’t show evident 89Zr-trastuzumab uptake, scan considered positive |
| 8 | Evaluation of HER2 status | Suspicious lymph node, no biopsy possible | Nodes | Nodes | None | No ^68^Ga-HER2 affibody uptake in the lymph nodes, which may be caused by excessive uptake of blood vessels around lymph nodes, scan considered negative |
| 9 | Heterogeneous HER2 status over time | HER2- primary breast cancer and HER2+ metastasis during course of disease | Liver, bone, nodes | Liver, bone, nodes | Liver, bone, nodes | Entire tumour load shows ^68^Ga-HER2 affibody uptake, scan considered positive |
| 10 | synchronous primary cancers | HER2+ primary breast cancer and HER2- secondary breast cancer | Brian, thyroid, subcutaneous, nodes | Thyroid, bone, subcutaneous, nodes | Thyroid, bone, nodes | Thyroid, bone and nodes metastases show ^68^Ga-HER2 affibody uptake, brain metastasis has undergone surgery and radiotherapy, scan considered positive |
| 11 | Heterogeneous HER2 status over time | HER2+ primary breast cancer and HER2- metastasis during course of disease | Lung, liver, bone | Lung, liver, pleura, bone, nodes | Lung, pleura, bone, nodes | Dominant part of tumour load shows ^68^Ga-HER2 affibody uptake, the background radioactivity of liver is too high to be evaluated, scan considered positive |
| 12 | Evaluation of HER2 status | Multiple lesions, no repeated biopsy possible | Bone | Bone | Bone | Bone metastasis shows ^68^Ga-HER2 affibody uptake, scan considered positive |
| 13 | synchronous primary cancers | HER2+ breast cancer and lung cancer | Liver, lung, bone, nodes | Liver, bone | Liver, bone | Liver and bone metastases show ^68^Ga-HER2 affibody uptake, scan considered positive |
| 14 | Evaluation of HER2 status | Liver metastases possible visualized on MRI | Bone | Bone | Bone | Bone metastasis shows ^68^Ga-HER2 affibody uptake, scan considered positive |
| 15 | Evaluation of HER2 status | Suspicious lung nodule, no biopsy possible | Lung | Lung | None | No focal ^68^Ga-HER2 affibody uptake, scan considered negative |
| 16 | Evaluation of HER2 status | Multiple lesions, no repeated biopsy possible | Chest wall, bone | Bone | Bone | Bone metastasis shows ^68^Ga-HER2 affibody uptake, chest wall metastasis has undergone radiotherapy, scan considered positive has |
| 17 | Evaluation of HER2 status | Multiple lesions, no repeated biopsy possible | Breast, liver, bone, nodes | Breast, liver, bone, nodes | None | No focal ^68^Ga-HER2 affibody uptake, scan considered negative |
| 18 | synchronous primary cancers | HER2+ primary breast cancer and HER2- secondary breast cancer | Bone, nodes | Bone, nodes | Bone, nodes | Dominant part of tumour load shows ^68^Ga-HER2 affibody uptake, scan considered positive |
| 19 | Evaluation of HER2 status | Doubtful whether continuation of anti-HER2 therapy is indicated after years of treatment and progression of disease | Liver, lung ,bone | Liver, bone | Liver, bone | Dominant part of tumour load shows ^68^Ga-HER2 affibody uptake, no ^68^Ga-HER2 affibody uptake in lung lesion, scan considered positive |
| 20 | Heterogeneous HER2 status over time | HER2+ primary breast cancer and HER2- metastasis during course of disease | Brian, chest wall, nodes | Chest wall, nodes | Brian, nodes | Brain metastasis has ^68^Ga-HER2 affibody uptake without ^18^F-FDG uptake, chest wall metastasis has ^18^F-FDG uptake without ^68^Ga-HER2 affibody uptake, scan considered equivocal |
| 21 | synchronous primary cancers | HER2+ breast cancer and oral cancer | Chest wall, lung, bone, nodes | Chest wall, lung, bone, nodes | Nodes | Only slight ^68^Ga-HER2 affibody uptake in a suspicious lymph node, scan considered negative |
| 22 | Evaluation of HER2 status | Doubtful whether continuation of anti-HER2 therapy is indicated after years of treatment and progression of disease | Chest wall, liver, lung, bone, nodes | Chest wall, liver, lung, bone, nodes | Chest wall, liver, bone, nodes | Dominant part of tumour load shows ^68^Ga-HER2 affibody uptake, no ^68^Ga-HER2affibody uptake in lung lesion, scan considered positive |
| 23 | synchronous primary cancers | HER2+ breast cancer and thyroid cancer | Nodes | Nodes | Nodes | Lymph node metastasis shows ^68^Ga-HER2 affibody uptake, scan considered positive |
| 24 | synchronous primary cancers | Suspicious lymph node, no biopsy possible | Nodes | Nodes | None | No focal ^68^Ga-HER2 affibody uptake, scan considered negative |
